# Anaemia is independently associated with mortality in patients with hepatocellular carcinoma

**DOI:** 10.1016/j.esmoop.2024.103593

**Published:** 2024-06-07

**Authors:** T. Meischl, L. Balcar, Y.-R. Park, L. Bucher, P. Meier, Y. Suhr, K. Pomej, M. Mandorfer, T. Reiberger, M. Trauner, B. Scheiner, M. Pinter

**Affiliations:** 1 Division of Gastroenterology und Hepatology, Department of Medicine III, Medical University of Vienna, Vienna; 2 Liver Cancer (HCC) Study Group Vienna, Division of Gastroenterology and Hepatology, Department of Medicine III, Medical University of Vienna, Vienna; 3 3^rd^ Medical Department (Haematology & Oncology), Hanusch-Krankenhaus, Vienna, Austria; 4 Department of Dermatology, University Hospital Regensburg, Regensburg, Germany; 5 Christian Doppler Laboratory for Portal Hypertension and Liver Fibrosis, Medical University of Vienna, Vienna, Austria

**Keywords:** liver cancer, hepatocellular carcinoma, HCC, anaemia

## Abstract

**Background:**

Anaemia is frequent in patients with cancer and/or liver cirrhosis and is associated with impaired quality of life. Here, we investigated the impact of anaemia on overall survival (OS) and clinical characteristics in patients with hepatocellular carcinoma (HCC).

**Materials and methods:**

HCC patients treated between 1992 and 2018 at the Medical University of Vienna were retrospectively analysed. Anaemia was defined as haemoglobin level <13 g/dl in men and <12 g/dl in women.

**Results:**

Of 1262 assessable patients, 555 (44.0%) had anaemia. The main aetiologies of HCC were alcohol-related liver disease (*n* = 502; 39.8%) and chronic hepatitis C (*n* = 375; 29.7%). Anaemia was significantly associated with impaired liver function, portal hypertension, more advanced Barcelona Clinic Liver Cancer stage and elevated C-reactive protein (CRP). In univariable analysis, anaemia was significantly associated with shorter median OS [9.5 months, 95% confidence interval (95% CI) 7.3-11.6 months] versus patients without anaemia (21.5 months, 95% CI 18.3-24.7 months) (*P* < 0.001). In multivariable analysis adjusted for age, Model for End-stage Liver Disease, number of tumour nodules, size of the largest nodule, macrovascular invasion, extrahepatic spread, first treatment line, alpha-fetoprotein and CRP, anaemia remained an independent predictor of mortality (adjusted hazard ratio 1.23, 95% CI 1.06-1.43, *P* = 0.006).

**Conclusions:**

Anaemia was significantly associated with mortality in HCC patients, independent of established liver- and tumour-related prognostic factors. Whether adequate management of anaemia can improve outcome of HCC patients needs further evaluation.

## Highlights

• Anaemia was associated with more advanced HCC stage and more severe underlying liver disease.
• Anaemia was independently associated with mortality in HCC patients.
• There was no survival difference between CTCAE v5.0 grade 1 vs. 2-4 and between micro- vs. normo- vs. macrocytic anaemia.

## Introduction

Liver cancer, of which hepatocellular carcinoma (HCC) represents the majority, is the sixth most frequent cancer and the third most common malignant cause of death worldwide.^1^ Mostly, it develops in a cirrhotic liver due to hepatitis B virus (HBV), hepatitis C virus (HCV), alcohol-related liver disease (ALD) or metabolic dysfunction-associated steatotic liver disease (MASLD).^2^

Several prognostic serum biomarkers have been proposed for patients suffering from HCC. While alpha-fetoprotein (AFP) and C-reactive protein (CRP) are amongst the most studied ones,^3^, ^4^, ^5^ the prognostic role of anaemia, a condition often found in cancer patients,^6^ is less clear. Anaemia is defined by the World Health Organization (WHO) as a haemoglobin level <13 g/dl in men and <12 g/dl in women.^7^ It can be stratified by mean corpuscular volume (MCV) into microcytic, normocytic and macrocytic anaemia which is suggestive of specific causes.^8^ In cancer patients, anaemia is a common comorbidity that is associated with worse outcome and impaired quality of life.^9^^,^^10^ One important reason for anaemia in malignant diseases is inflammation, leading to anaemia of chronic disease, mainly caused by a dysfunctional iron metabolism.^11^ Further mechanisms, e.g. chronic bleeding, haemolysis, malnutrition or bone marrow infiltration, may also play a role in certain types of cancer.^9^ In patients with liver cirrhosis and portal hypertension, anaemia is also frequently present due to various risk factors such as chronic inflammation, gastrointestinal blood loss, malnutrition or destruction of red blood cells, and its severity correlates with hepatic dysfunction.^12^, ^13^, ^14^

Although HCC patients suffer from two conditions commonly accompanied by anaemia (i.e. liver cirrhosis and cancer), direct evidence about the impact of anaemia on their survival is sparse. Two studies found a correlation between low haemoglobin levels and overall survival (OS) in small cohorts of HCC patients.^15^^,^^16^ Low haemoglobin levels were also associated with diminished survival in cohorts treated with programmed cell death protein 1 (PD-1) inhibitors^17^ and Yttrium-90 radioembolisation.^18^ By using computational intelligence methods, a recent study identified haemoglobin levels as one of the most predictive factors for survival of HCC patients although it remains unclear which treatment the patients received.^19^

Here, we conducted a retrospective analysis of the impact of anaemia on OS and clinical characteristics in a large, well-characterised cohort of HCC patients.

## Materials and Methods

### Patients

All adult patients who were diagnosed with HCC at the Medical University of Vienna from 01 August 1992 to 30 April 2018 were included. Diagnosis of HCC was based on histology or radiology in accordance with the European Association for the Study of the Liver (EASL) guidelines in their most current version.^20^, ^21^, ^22^ Patients with missing haemoglobin level and/or insufficient follow-up were excluded from this analysis.

### Data collection and definition of variables

All data were collected retrospectively from the patients’ electronic health records. If not stated otherwise, the values at the time of HCC diagnosis were collected. All laboratory parameters recorded for this study were measured in the ISO-certified laboratory of the Medical University of Vienna. Child–Pugh score and Model for End-stage Liver Disease (MELD) score were used to describe liver function. Tumour staging was defined according to the Barcelona Clinic Liver Cancer (BCLC) classification.^23^ The presence of portal hypertension was assumed in all patients with oesophageal/gastric varices, portal-hypertensive gastropathy, ascites, a hepatic venous pressure gradient of ≥10 mmHg or a liver stiffness measurement value measured by vibration-controlled transient elastography ≥25 kPa.^24^ The Milan criteria as a summative parameter for tumour extent were defined according to Mazzaferro et al.^25^ (one tumour nodule ≤5 cm or ≤3 nodules ≤3 cm, no macrovascular invasion, no extrahepatic manifestation).

Anaemia was defined according to the WHO definition,^7^ i.e. a haemoglobin level below 13 g/dl in men and below 12 g/dl in women. The severity of anaemia was stratified into four grades according to the Common Terminology Criteria of Adverse Events (CTCAE) version 5.0.^26^ Anaemia type was stratified by MCV into microcytic (<80 fl), normocytic (80-96 fl) and macrocytic anaemia (>96 fl).

The definition of systemic treatment in the context of this manuscript includes sorafenib, lenvatinib, regorafenib, cabozantinib, ramucirumab and the immune checkpoint inhibitors nivolumab, pembrolizumab as well as the combination of atezolizumab and bevacizumab. All other experimental systemic treatments (e.g. thalidomide, octreotide, etc.) were included in the ‘other treatment’ group. For the purpose of statistical analysis, liver transplantation, surgical resection and local ablation were defined as curative treatments, whereas transarterial chemoembolisation (TACE), systemic therapy, best supportive care (BSC) and all other treatments were defined as palliative treatments.

### Definition of endpoints and statistical analysis

Baseline patient characteristics of the overall study population and the subgroups anaemia versus no anaemia are presented using descriptive statistics. The association of categorical variables was tested by chi-square test. Means of continuous variables were compared by *t*-test.

OS was defined as the time from the initiation of the first treatment line until the date of death. If a patient did not receive any treatment, the date of diagnosis was defined as the starting point of OS. Patients who had still been alive at 31 October 2021 (end of follow-up) were censored at the time of last contact. Median follow-up was calculated by the reverse Kaplan–Meier method.

Survival plots were calculated by the Kaplan–Meier method and compared by log-rank test (univariable analysis). Multivariable survival analysis was done by Cox regression. Variables were included into the multivariable model if they were statistically significant predictors of OS in univariable analysis. If two or more variables showed a strong correlation (e.g. Child–Pugh class and MELD score), only one of these variables was inserted into the multivariable model in order to avoid collinearity.

In general, a *P* value < 0.05 was considered statistically significant. Wherever appropriate, the level of statistical significance was corrected by using the Bonferroni method—in this case, the corrected level of statistical significance is given in the footnote of the respective table. Statistical analyses were carried out using SPSS version 29.0 (Chicago, IL) and R 4.3.1 (R Core Team, R Foundation for Statistical Computing, Vienna, Austria). Receiver operating characteristic (ROC) curves for the outcomes of interest were calculated using the ‘proc’ package of R.

### Ethical considerations

This retrospective analysis was approved by the local ethics committee of the Medical University of Vienna (reference number 1759/2015). As this study is a retrospective analysis of anonymised patient data, the obligation to obtain written informed consent was waived by the ethics committee.

## Results

### Patient characteristics

Of 1415 HCC patients, 152 were a priori excluded from analysis due to missing haemoglobin levels and one because of insufficient follow-up. Thus, 1262 patients were analysed (Supplementary Figure S1, available at https://doi.org/10.1016/j.esmoop.2024.103593).

Baseline patient characteristics are presented in Table 1. In the overall study population, the mean age was 63.7 years [standard deviation (SD) ± 10.1 years] and the majority of patients were male (*n* = 1044, 82.7%). Most patients (*n* = 1102, 87.7%) had liver cirrhosis. The leading aetiologies were ALD (*n* = 502, 39.8%), HCV (*n* = 375, 29.7%), HBV (*n* = 118, 9.4%) and MASLD (*n* = 102, 8.1%). The first treatment line was liver transplantation in 51 patients (4.0%), surgical resection in 117 (9.3%), local ablation in 241 (19.1%), TACE in 317 (25.1%), systemic therapy in 141 (11.2%), other treatments in 175 (13.9%) and BSC in 220 (17.4%).

**Table 1:** Patient characteristics

Total		Overall study population		Anaemia		No anaemia		*P* value (chi-square test)
		*N*	% of total	*N*	%	*N*	%	—
		1262	100.0	555	44.0	707	56.0	
Variable	Category							
Age (years) (*n* = 1262)	≥65	592	46.9	260	46.8	332	47.0	0.968
	<65	670	53.1	295	53.2	375	53.0	
Sex (*n* = 1262)	Male	1044	82.7	470	84.7	574	81.2	0.103
	Female	218	17.3	85	15.3	133	18.8	
Aetiology (*n* = 1262)	Alcohol	502	39.8	249	44.9	253	35.8	0.027
	HCV	375	29.7	154	27.7	221	31.3	
	HBV	118	9.4	47	8.5	71	10.0	
	MASLD	102	8.1	41	7.4	61	8.6	
	Unknown/other	165	13.1	64	11.5	101	14.3	
Liver cirrhosis (*n* = 1256)	Yes	1102	87.7	501	90.6	601	85.5	0.006
	No	154	12.3	52	9.4	102	14.5	
Child–Pugh class (*n* = 1218)	A (score 5 or 6)	579	47.5	164	30.7	415	60.8	<0.001
	B (score 7-9)	438	36.0	239	44.7	199	29.1	
	C (score 10 or higher)	201	16.5	132	24.7	69	10.1	
MELD score (*n* = 1241)	6-8	340	27.4	98	17.9	242	35.0	<0.001
	8-18	786	63.3	367	66.8	419	60.5	
	≥19	115	9.3	84	15.3	31	4.5	
Portal hypertension (*n* = 1255)	Present	884	70.4	449	81.5	435	61.8	<0.001
	Absent	371	29.6	102	18.5	269	38.2	
Number of nodules (*n* = 1257)	1	589	46.9	257	46.7	332	47.0	0.996
	2-3	385	30.6	169	30.7	216	30.6	
	>3	283	22.5	124	22.5	159	22.5	
Size of the largest tumour nodule (cm) (*n* = 1247)	≤3	319	25.6	136	25.0	183	26.1	0.320
	>3 and ≤5	353	28.3	145	26.6	208	29.6	
	>5	575	46.1	264	48.4	311	44.3	
Macrovascular invasion (*n* = 1225)	Present	262	21.4	131	24.5	131	19.0	0.018
	Absent	963	78.6	403	75.5	560	81.0	
Extrahepatic manifestation (*n* = 1202)	Present	138	11.5	73	14.0	65	9.6	0.018
	Absent	1064	88.5	450	86.0	614	90.4	
Milan in/out (*n* = 1237)	Milan in	370	29.9	152	28.3	218	31.1	0.280
	Milan out	867	70.1	385	71.7	482	68.9	
BCLC stage (*n* = 1250)	0	59	4.7	19	3.4%	40	5.7	<0.001
	A	331	26.5	111	20.1	220	31.5	
	B	206	16.5	63	11.4	143	20.5	
	C	423	33.8	203	36.8	220	31.5	
	D	231	18.5	156	28.3	75	10.7	
First treatment line—curative versus palliative (*n* = 1262)	Curative	409	32.4	150	27.0	259	36.6	<0.001
	Palliative	853	67.6	405	73.0	448	63.4	
First treatment line (*n* = 1262)	Liver transplant	51	4.0	31	5.6	20	2.8	<0.001
	Resection	117	9.3	28	5.0	89	12.6	
	Local ablation	241	19.1	91	16.4	150	21.2	
	TACE	317	25.1	125	22.5	192	27.2	
	Systemic treatment	141	11.2	62	11.2	79	11.2	
	Other	175	13.9	77	13.9	98	13.9	
	Best supportive care	220	17.4	141	25.4	79	11.2	
AFP (ng/ml) (*n* = 1213)	<100	728	60.0	311	58.1	417	61.5	0.278
	100-400	139	11.5	59	11.0	80	11.8	
	400-1000	101	8.3	53	9.9	48	7.1	
	>1000	245	20.2	112	20.9	133	19.6	
CRP (mg/dl) (*n* = 1188)	<1	611	51.4	204	38.9	407	61.3	<0.001
	≥1	577	48.6	320	61.1	257	38.7	

Level of statistical significance (corrected with the Bonferroni method): 0.0029.

AFP, alpha-fetoprotein; BCLC, Barcelona Clinic Liver Cancer; CRP, C-reactive protein; HBV, hepatitis B virus; HCV, hepatitis C virus; MASLD, metabolic dysfunction-associated steatotic liver disease; MELD, Model for End-stage Liver Disease; TACE, transarterial chemoembolisation.

### Association between anaemia and other patient characteristics

At diagnosis, 555 patients (44.0%) had anaemia. Mean haemoglobin level was 12.9 g/dl (SD ± 2.0) in the total cohort, 11.1 g/dl (SD ± 1.3) in patients with anaemia and 14.3 g/dl (SD ± 1.1) in patients without anaemia (Table 2). Of the patients with anaemia (*n* = 555), 70 (12.6%) patients had microcytic anaemia, 375 (67.6%) had normocytic anaemia and 110 (19.8%) had macrocytic anaemia.

**Table 2:** Patient characteristics with focus on anaemia-related characteristics

Total		Overall study population		Anaemia		No anaemia		*P* value (chi-square test/*t*-test)
		*N*	% of total	*N*	%	*N*	%	
		1262	100.0%	555	44.0%	707	56.0%	—
Haemoglobin level (g/dl) (*n* = 1262)	Mean (±SD)	12.9 ± 2.0		11.1 ± 1.3		14.3 ± 1.1		—
Anaemia (*n* = 1262)	No	707	56.0%	0	0.0%	707	100.0%	—
	CTCAE v5.0 grade 1 (Hb ≥10 g/dl)	459	36.4%	459	82.7%	0	0.0%	
	CTCAE v5.0 grade 2-4 (Hb <10 g/dl)	96	7.6%	96	17.3%	0	0.0%	
Erythrocyte number (*n* = 1262)	Mean (±SD)	4.18 ± 0.63		3.72 ± 0.52		4.54 ± 0.45		<0.001
	Normal	296	23.5%	25	4.5%	271	38.3%	<0.001
	Below LLN	966	76.5%	530	95.5%	436	61.7%	
Haematocrit (%) (*n* = 1261)	Mean (±SD)	37.7 ± 5.3		33.0 ± 3.6		41.3 ± 3.2		<0.001
	Normal	339	26.9%	2	0.4%	337	47.7%	<0.001
	Below LLN	922	73.1%	553	99.6%	369	52.3%	
Anaemia, stratified by MCV (*n* = 1262)	No anaemia	707	56.0%	0	0.0%	707	100.0%	—
	Microcytic, MCV <80 fl	70	5.5%	70	12.6%	0	0.0%	
	Normocytic, MCV 80-96 fl	375	29.7%	375	67.6%	0	0.0%	
	Macrocytic, MCV >96 fl	110	8.7%	110	19.8%	0	0.0%	
Serum ferritin level (μg/l) (*n* = 830)	Mean (±SD)	531.3 ± 1186.3		527.8 ± 1458.2		534.0 ± 925.9		0.944
	<20 μg/l	33	4.0%	25	6.9%	8	1.7%	<0.001
	20-200 μg/l	548	66.0%	227	62.9%	321	68.4%	
	>200 μg/l	249	30.0%	109	30.2%	140	29.9%	
Serum transferrin level (mg/dl) (*n* = 867)	Mean (±SD)	232.8 ± 65.5		228.6 ± 74.0		236.0 ± 58.2		0.112
	<200 mg/dl	278	32.1%	148	39.6%	130	26.4%	<0.001
	≥200 mg/dl	589	67.9%	226	60.4%	363	73.6%	
Transferrin saturation (%) (*n* = 846)	Mean (±SD)	34.2 ± 23.7		28.8 ± 23.6		38.2 ± 22.9		<0.001
	<16%	206	24.3%	142	39.2%	64	13.2%	<0.001
	16%-45%	427	50.5%	149	41.1%	278	57.4%	
	>45%	213	25.2%	71	19.6%	142	29.3%	
History of variceal bleeding (*n* = 1101)	Yes	636	57.8%	336	67.9%	300	49.5%	<0.001
	No	465	42.2%	159	32.1%	306	50.5%	
INR (*n* = 1241)	Mean (±SD)	1.26 ± 0.31		1.31 ± 0.34		1.22 ± 0.28		<0.001
	Normal (<1.5)	1074	86.5%	449	81.8%	625	90.3%	<0.001
	High (≥1.5)	167	13.5%	100	18.2%	67	9.7%	
Platelet count (*n* =1261)	Mean (±SD)	156.5 ± 95.1		160.5 ± 111.8		153.4 ± 79.5		0.209
	≥100 G/l	896	71.1%	370	66.8%	526	74.4%	0.003
	<100 G/l	365	28.9%	184	33.2%	181	25.6%	
Anticoagulation (*n* = 1257)	Yes	101	8.0%	48	8.7%	53	7.5%	0.446
	No	1156	92.0%	504	91.3%	652	92.5%	
Anti-platelet therapy (*n* = 1257)	Yes	133	10.6%	55	10.0%	78	11.1%	0.529
	No	1124	89.4%	497	90.0%	627	88.9%	
NSBB (*n* = 1256)	Yes	291	23.2%	160	29.0%	131	18.6%	<0.001
	No	965	76.8%	392	71.0%	573	81.4%	
PPI (*n* = 1256)	Yes	489	38.9%	260	47.2%	229	32.5%	<0.001
	No	767	61.1%	291	52.8%	476	67.5%	

Level of statistical significance (corrected with the Bonferroni method): 0.0026.

CTCAE, Common Terminology Criteria of Adverse Events version 5.0; Hb, haemoglobin; INR, international normalised ratio; LLN, lower limit of normal; MCV, mean corpuscular volume; NSBB, non-selective beta-blocker; PPI, proton pump inhibitor; SD, standard deviation.

Anaemia was significantly associated with impaired liver function (Child–Pugh stage, *P* < 0.001; MELD score, *P* < 0.001), portal hypertension (*P* < 0.001), more advanced BCLC stage (*P* < 0.001), palliative first-line treatment (*P* < 0.001), elevated CRP level (*P* < 0.001) (Table 1), low serum ferritin level (*P* < 0.001), low serum transferrin level (*P* < 0.001), low transferrin saturation (*P* < 0.001), history of variceal bleeding (*P* < 0.001), high international normalised ratio (*P* < 0.001), non-selective beta-blocker intake (*P* < 0.001) and proton pump inhibitor intake (*P* < 0.001) (Table 2).

### Association between anaemia and overall survival

Median estimated follow-up was 93.6 months. Of 1262 patients analysed, 973 (77.1%) died. The median overall survival (mOS) of the total study cohort was 14.8 months [95% confidence interval (CI) 12.9-16.7 months; Table 3].

**Table 3:** Univariable overall survival analysis

Total		*N*	mOS (months)	95% CI	*P* value (log-rank)
		1262	14.8	12.9-16.7	—
Variable	Category				
Age (years) (*n* = 1262)	≥65	592	14.2	12.0-16.4	<0.001
	<65	670	17.9	14.3-21.5	
Sex (*n* = 1262)	Male	1044	14.9	12.9-16.9	0.952
	Female	218	14.4	10.1-18.7	
Aetiology (*n* = 1262)	Alcohol	502	12.7	10.5-14.9	0.008
	HCV	375	19.3	15.4-23.2	
	HBV	118	9.3	1.0-17.6	
	MASLD	102	23.6	9.6-37.6	
	Unknown/other	165	14.5	10.1-18.8	
Anaemia (*n* = 1262)	No	707	21.5	18.3-24.7	<0.001
	Yes	555	9.5	7.3-11.6	
Anaemia—CTCAE grade (*n* = 1262)	No anaemia	707	21.5	18.3-24.7	<0.001
	Anaemia grade 1	459	9.3	6.9-11.7	
	Anaemia grade 2-4	96	10.3	6.3-14.4	
Anaemia—CTCAE v5.0 grade, excluding all patients without anaemia from the analysis (*n* = 555)	Anaemia grade 1	459	9.3	6.9-11.7	0.953
	Anaemia grade 2-4	96	10.3	6.3-14.4	
Anaemia, stratified by MCV (*n* = 1262)	No anaemia	707	21.5	18.3-24.7	<0.001
	Microcytic, MCV <80 fl	70	13.9	5.2-22.7	
	Normocytic, MCV 80-96 fl	375	9.4	6.9-11.9	
	Macrocytic, MCV >96 fl	110	7.6	4.2-11.0	
Anaemia, stratified by MCV, excluding all patients without anaemia from the analysis (*n* = 555)	Microcytic, MCV < 80 fl	70	13.9	5.2-22.7	0.396
	Normocytic, MCV 80-96 fl	375	9.4	6.9-11.9	
	Macrocytic, MCV > 96 fl	110	7.6	4.2-11.0	
Liver cirrhosis (*n* = 1256)	No	154	20.0	13.3-26.7	0.045
	Yes	1102	14.3	12.4-16.2	
Child–Pugh class (*n* = 1218)	A (score 5 or 6)	579	25.7	21.3-30.1	<0.001
	B (score 7-9)	438	11.0	9.0-13.0	
	C (score ≥ 10)	201	3.8	2.4-5.2	
MELD score (*n* = 1241)	6-8	340	29.1	22.8-35.3	<0.001
	8-18	786	13.9	12.0-15.8	
	≥19	115	1.9	1.0-2.7	
Portal hypertension (*n* = 1255)	Present	884	10.9	9.3-12.5	<0.001
	Absent	371	30.7	24.3-37.1	
Number of nodules (*n* = 1257)	1	589	26.2	19.3-33.0	<0.001
	2-3	385	13.8	10.7-16.8	
	>3	283	6.7	5.2-8.3	
Size of the largest tumour nodule (cm) (*n* = 1247)	≤3	319	41.0	31.2-50.9	<0.001
	>3 and ≤5	353	19.0	14.7-23.3	
	>5	575	8.2	6.6-9.8	
Macrovascular invasion (*n* = 1225)	Absent	963	20.0	17.3-22.8	<0.001
	Present	262	4.3	0.5-3.3	
Extrahepatic manifestation (*n* = 1202)	Absent	1064	18.1	15.8-20.4	<0.001
	Present	138	3.7	2.9-4.5	
Milan in/out (*n* = 1237)	Milan in	370	47.7	39.2-56.2	<0.001
	Milan out	867	9.5	8.0-10.9	
BCLC stage (*n* = 1250)	0-A	390	47.7	40.7-54.8	<0.001
	B	206	18.7	16.3-21.1	
	C	423	6.5	5.1-7.9	
	D	231	3.5	2.3-4.7	
First treatment line—curative versus palliative (*n* = 1262)	Curative	409	41.4	33.3-49.6	<0.001
	Palliative	853	9.3	7.9-10.8	
First treatment line (*n* = 1262)	Liver transplant	51	30.3	0.0-64.8	<0.001
	Resection	117	71.5	49.0-94.1	
	Local ablation	241	32.6	24.5-40.6	
	TACE	317	23.6	18.8-28.4	
	Systemic treatment	141	8.0	6.1-9.9	
	Other	175	7.0	4.2-9.8	
	Best supportive care	220	2.3	1.8-2.8	
AFP (ng/ml) (*n* = 1213)	<100	728	23.0	19.2-26.9	<0.001
	100-400	139	12.8	8.5-17.1	
	400-1000	101	11.9	7.1-16.8	
	>1000	245	4.1	3.2-5.0	
AFP (ng/ml) (*n* = 1213)	<1000	968	19.2	16.6-21.9	<0.001
	≥1000	245	4.1	3.2-5.0	
CRP (mg/dl) (*n* = 1188)	<1	611	31.0	25.6-36.4	<0.001
	≥1	577	6.5	5.1-7.8	

Level of statistical significance (corrected with the Bonferroni method): 0.0022.

AFP, alpha-fetoprotein; BCLC, Barcelona Clinic Liver Cancer; CI, confidence interval; CRP, C-reactive protein; CTCAE v5.0, Common Terminology Criteria of Adverse Events version 5.0; HBV, hepatitis B virus; HCV, hepatitis C virus; MASLD, metabolic dysfunction-associated steatotic liver disease; MCV, mean corpuscular volume; MELD, Model for End-stage Liver Disease; mOS, median overall survival; TACE, transarterial chemoembolisation.

**Figure 1:**
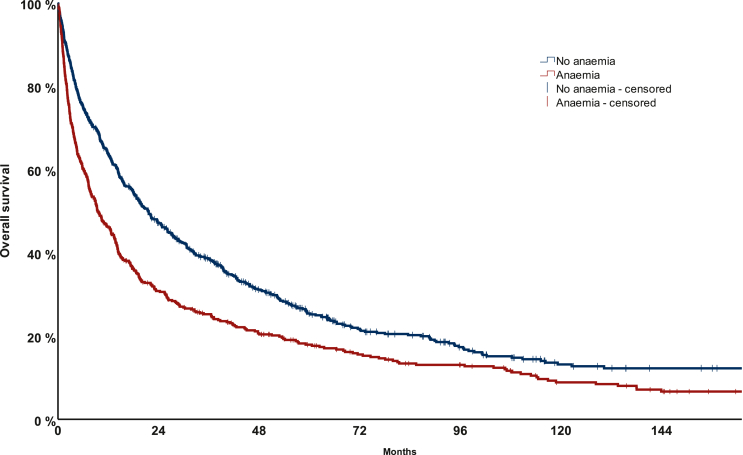
Overall survival (OS) according to the presence or absence of anaemia.

In univariable analysis, anaemia was associated with a shorter mOS (9.5 months, 95% CI 7.3-11.6 months), in comparison to patients without anaemia (21.5 months, 95% CI 18.3-24.7 months) (*P* < 0.001; Figure 1; Table 3). There was no significant difference between patients with CTCAE v5.0 grade 1 anaemia (mOS 9.3 months, 95% CI 6.9-11.7 months), compared to patients with grade 2-4 anaemia (mOS 10.3 months, 95% CI 6.3-14.4 months) (*P* = 0.953; Supplementary Figure S2, available at https://doi.org/10.1016/j.esmoop.2024.103593; Table 3). Furthermore, there was no significant difference between patients with microcytic anaemia (mOS 13.9 months, 95% CI 5.2-22.7 months), normocytic anaemia (mOS 9.4 months, 95% CI 6.9-11.9 months) and macrocytic anaemia (mOS 7.6 months, 95% CI 4.2-11.0 months) (*P* = 0.396; Supplementary Figure S3, available at https://doi.org/10.1016/j.esmoop.2024.103593; Table 3).

Further characteristics significantly associated with OS in univariable analysis included age (≥65 versus <65 years; *P* < 0.001), Child–Pugh class (*P* < 0.001), MELD score (*P* < 0.001), portal hypertension (*P* < 0.001), number of tumour nodules (*P* < 0.001), size of the largest nodule (*P* < 0.001), macrovascular invasion (*P* < 0.001), extrahepatic manifestation (*P* < 0.001), the Milan criteria (in versus out; *P* < 0.001), BCLC stage (*P* < 0.001), type of first-line treatment (*P* < 0.001), AFP (*P* < 0.001) and CRP (*P* < 0.001) (Table 3). The association of anaemia with OS in different BCLC stages is shown in Supplementary Table S1, available at https://doi.org/10.1016/j.esmoop.2024.103593.

In multivariable analysis, anaemia was significantly associated with mortality [adjusted hazard ratio (aHR) 1.23, 95% CI 1.06-1.43, *P* = 0.006; Table 4]. Age (continuous), MELD score (continuous), number of tumour nodules (1 versus 2-3 versus >3), size of the largest nodule (≤3 versus 3-5 versus >5 cm), macrovascular invasion, extrahepatic manifestation, first treatment line (curative versus palliative), AFP (<1000 versus ≥1000) and CRP (<1 versus ≥1) were significantly associated with OS in multivariable analysis as well (Table 4). Furthermore, haemoglobin level as a continuous variable was significantly associated with mortality (aHR 0.96, 95% CI 0.92-0.99, *P* = 0.027; Supplementary Table S2, available at https://doi.org/10.1016/j.esmoop.2024.103593), independent of various well-known risk factors. Prognostic ROC curves of AFP and anaemia were similar (Supplementary Figure S4, available at https://doi.org/10.1016/j.esmoop.2024.103593).

**Table 4:** Multivariable overall survival analysis

Variable	Category	HR	95% CI	*P* value
Anaemia	No	1.00	**—**	0.006
	Yes	1.23	1.06-1.43	
Age (years)	Continuous	1.01	1.01-1.02	<0.001
MELD score	Continuous	1.04	1.02-1.05	<0.001
Number of nodules	1	1.00	**—**	<0.001
	2-3	1.30	1.11-1.54	
	>3	1.57	1.30-1.89	
Size of the largest tumour nodule (cm)	≤3	1.00	**—**	<0.001
	>3 and ≤5	1.34	1.10-1.64	
	>5	1.70	1.39-2.06	
Macrovascular invasion	Absent	1.00	**—**	<0.001
	Present	1.40	1.16-1.69	
Extrahepatic manifestation	Absent	1.00	**—**	0.002
	Present	1.43	1.15-1.79	
First treatment line	Curative	1.00	**—**	<0.001
	Palliative	1.45	1.23-1.72	
AFP (ng/ml)	<1000	1.00	**—**	<0.001
	≥1000	1.64	1.37-1.96	
CRP (mg/dl)	<1	1.00	**—**	<0.001
	≥1	1.58	1.35-1.85	

AFP, alpha-fetoprotein; CI, confidence interval; CRP, C-reactive protein; HR, hazard ratio; MELD, Model for End-stage Liver Disease.

## Discussion

In this large retrospective study, we identified anaemia as a significant comorbidity in HCC patients which is associated with tumour stage, more advanced liver function impairment and inflammation, and negatively impacts patients’ outcome. A key strength of our study is the large number of well-characterised patients with a long follow-up, allowing for meaningful and robust survival analysis. Our results are in line with other, albeit small, studies that have also reported a link between haemoglobin levels and impaired survival in HCC patients treated with PD-1 inhibitors,^17^ Yttrium-90 radioembolisation^18^ and various first-line treatments.^15^^,^^16^

Anaemia is usually stratified by MCV into micro-, normo- and macrocytic anaemia, which is closely linked to possible causes of anaemia.^8^^,^^9^ In our cohort, most anaemic patients suffered from normocytic anaemia, suggesting that inflammation-driven—so-called ‘anaemia of chronic disease’ (or more recently termed ‘anaemia of inflammation’)—was the most common cause. This is a comorbidity that is frequently found in cancer patients.^11^

Beside tumour stage (i.e. BCLC stage), Child–Pugh class, MELD score and portal hypertension were significantly associated with anaemia, underlining that impaired liver function and splenomegaly/hypersplenism due to portal hypertension also contribute to the development of anaemia in HCC patients. Accordingly, in a cohort of patients with advanced chronic liver disease and portal hypertension, most anaemic patients had normocytic anaemia, and the presence of anaemia and lower haemoglobin levels were associated with deteriorating liver function.^12^ However, given that anaemia and haemoglobin level as a continuous variable were both significantly associated with mortality independent of MELD score in the multivariable model, we conclude that anaemia *per se* should be considered as an independent risk factor for mortality and not only as a surrogate marker for hepatic dysfunction and more advanced tumour stages.

CRP is a well-known marker for inflammation as well as more aggressive tumour biology and highly predictive for survival and other outcome parameters in HCC patients.^3^^,^^4^^,^^27^^,^^28^ Its significant association with anaemia in our cohort highlights the well-known and important role of inflammation in the development of anaemia.^11^

Possible limitations of our study include the retrospective design with all its potential biases and the long period of inclusion, possibly introducing heterogeneity in the diagnostic approach and standard of care. Moreover, some variables required to determine the cause of anaemia (e.g. folic acid, vitamin B12) were not routinely measured. Additionally, we assumed that all patients with ascites had portal hypertension; however, ascites in HCC patients may have other causes (e.g. hypoalbuminaemia, malignant ascites) and thus a small number of patients may have been misclassified as having portal hypertension.

It is noteworthy that cut-offs for haemoglobin levels for clinical decision making (e.g. for clinical trial inclusion, transfusion, erythropoietin administration)^29^, ^30^, ^31^ are generally lower than the cut-off defining anaemia according to WHO. In our cohort, we observed no OS difference in patients with mild (CTCAE grade 1) versus moderate/severe (grade 2-4) anaemia, and both groups had a markedly reduced OS compared to non-anaemic HCC patients. Whether targeting anaemia at earlier stages may be beneficial in terms of quality of life and survival of HCC patients requires prospective evaluation.

In conclusion, anaemia was significantly associated with worse OS in HCC patients, independent of various well-known prognostic factors including severity of liver function impairment. Furthermore, our data suggest a role of more advanced tumour stage, more severe liver function impairment and systemic inflammation in the multifactorial genesis of anaemia in patients with HCC. Prospective studies are needed to investigate potential benefits of measures to ameliorate anaemia in patients with HCC.

## Funding

None declared.

## Disclosure

**TM** received speaker honoraria from AstraZeneca, Chiesi and Janssen-Cilag; consulting fees from CSL Behring; and travel support from AstraZeneca, BeiGene, CSL Behring, Chiesi, Jazz Pharmaceuticals, Janssen-Cilag, Novartis and teva-ratiopharm. **LBa** received speaker honoraria from Chiesi. **MM** served as a speaker and/or consultant and/or advisory board member for AbbVie, Collective Acumen, Gilead and W. L. Gore & Associates; and received travel support from AbbVie and Gilead. **TR** received grant support from AbbVie, Boehringer Ingelheim, Gilead, Intercept/Advanz Pharma, MSD, Myr Pharmaceuticals, Philips Healthcare, Pliant, Siemens and W. L. Gore & Associates; speaking honoraria from AbbVie, Gilead, Intercept/Advanz Pharma, Roche, MSD, W. L. Gore & Associates; consulting/advisory board fees from AbbVie, AstraZeneca, Bayer, Boehringer Ingelheim, Gilead, Intercept/Advanz Pharma, MSD, Resolution Therapeutics and Siemens; and travel support from AbbVie, Boehringer Ingelheim, Dr. Falk Pharma, Gilead and Roche. **MT** received speaker fees from Bristol-Myers Squibb (BMS), Falk Foundation, Gilead, Intercept and Merck Sharp & Dohme (MSD); advisory board fees from AbbVie, Albireo, Boehringer Ingelheim, BiomX, Falk Pharma GmbH, GENFIT, Gilead, Hightide, Intercept, Janssen, MSD, Novartis, Phenex, Regulus and Shire; travel grants from AbbVie, Falk, Gilead and Intercept; and research grants from Albireo, Alnylam, CymaBay, Falk, Gilead, Intercept, MSD, Takeda and Ultragenyx. He is also a co-inventor of patents on the medical use of norUDCA filed by the Medical University of Graz. **BS** received grant support from AstraZeneca and Eisai; speaker honoraria from Eisai as well as travel support from AbbVie, AstraZeneca, Ipsen and Gilead. **MP** received speaker honoraria from Bayer, BMS, Eisai, Ipsen, Lilly, MSD and Roche; he is a consultant/advisory board member for AstraZeneca, Bayer, BMS, Eisai, Ipsen, Lilly, MSD and Roche; he received grants from Roche and BMS; he received travel support from Bayer, BMS, Ipsen and Roche. All other authors have declared no conflicts of interest.

## Data Sharing

Datasets are available from the corresponding author upon reasonable request.
