# Supplementary material for: Anaemia is independently associated with mortality in patients with hepatocellular carcinoma
Source: ESMO Open. 2024 Jun 7;9(6):103593. doi: 10.1016/j.esmoop.2024.103593 (PMC11214999; doi:10.1016/j.esmoop.2024.103593)
Supplement: Supplementary Material [file mmc1.docx]

Supplementary Material

| **Supplementary Table 1. Association of anaemia with overall survival in different BCLC stages.** | | | | | |
| --- | --- | --- | --- | --- | --- |
|  | | N | mOS | 95% CI | P value (log rank) |
| Total* | | 1250 | 14.6 | 12.8 - 16.4 | - |
| **BCLC stage** | **Anaemia** |  | | | |
| All BCLC stages* (n = 1250) | No | 698 | 20.8 | 17.4 - 24.2 | < 0.001 |
|  | Yes | 552 | 9.4 | 7.3 - 11.6 |  |
| BCLC stage 0 (n = 59) | No | 40 | 119.2 | 78.3 - 160.2 | 0.007 |
|  | Yes | 19 | 42.6 | 19.2 - 65.9 |  |
| BCLC stage A (n = 331 ) | No | 220 | 43.1 | 35.5 - 50.7 | 0.404 |
|  | Yes | 111 | 41.8 | 20.7 - 62.9 |  |
| BCLC stage B (n = 206) | No | 143 | 20.8 | 15.8 - 25.8 | 0.066 |
|  | Yes | 63 | 14.5 | 13.0 - 16.0 |  |
| BCLC stage C (n = 423) | No | 220 | 7.8 | 5.4 - 10.2 | 0.042 |
|  | Yes | 203 | 5.5 | 3.7 - 7.3 |  |
| BCLC stage D (n = 231) | No | 75 | 3.1 | 0.5 - 5.7 | 0.870 |
|  | Yes | 156 | 3.5 | 2.1 - 4.9 |  |
| *Patients in whom BCLC stage was not available (n = 12) were excluded from this analysis.  Abbreviations: BCLC, Barcelona Clinic Liver Cancer; CI, Confidence Interval; mOS, median overall survival.  Level of statistical significance (corrected with Bonferroni method): 0.0083 | | | | | |

| **Supplementary Table 2. Multivariable overall survival analysis with haemoglobin as a continuous variable.** | | | | |
| --- | --- | --- | --- | --- |
| Variable | Category | HR | 95% CI | P value |
| Haemoglobin level (g/dL) | continuous | 0.96 | 0.92 - 0.99 | 0.027 |
| Age (years) | continuous | 1.01 | 1.01 - 1.02 | < 0.001 |
| MELD score | continuous | 1.04 | 1.02 - 1.05 | < 0.001 |
| Number of nodules | 1 | 1.00 | - | < 0.001 |
|  | 2 - 3 | 1.30 | 1.10 - 1.53 |  |
|  | > 3 | 1.56 | 1.30 - 1.88 |  |
| Size of the largest tumour nodule (cm) | ≤ 3 | 1.00 | - | < 0.001 |
|  | > 3 and ≤ 5 | 1.36 | 1.11 - 1.66 |  |
|  | > 5 | 1.71 | 1.40 - 2.08 |  |
| Macrovascular invasion | Absent | 1.00 | - | < 0.001 |
|  | Present | 1.40 | 1.16 - 1.69 |  |
| Extrahepatic manifestation | Absent | 1.00 | - | < 0.001 |
|  | Present | 1.47 | 1.18 - 1.83 |  |
| First treatment line | Curative | 1.00 | - | < 0.001 |
|  | Palliative | 1.45 | 1.23 - 1.71 |  |
| AFP (ng/mL) | < 1000 | 1.00 | - | < 0.001 |
|  | ≥ 1000 | 1.60 | 1.34 - 1.92 |  |
| CRP (mg/dL) | < 1 | 1.00 | - | < 0.001 |
|  | ≥ 1 | 1.58 | 1.35 - 1.86 |  |
| Abbreviations: AFP, Alpha-Fetoprotein; CI, Confidence Interval; CRP, C-reactive protein; HR, Hazard Ratio; MELD, Model for End-stage Liver Disease. | | | | |


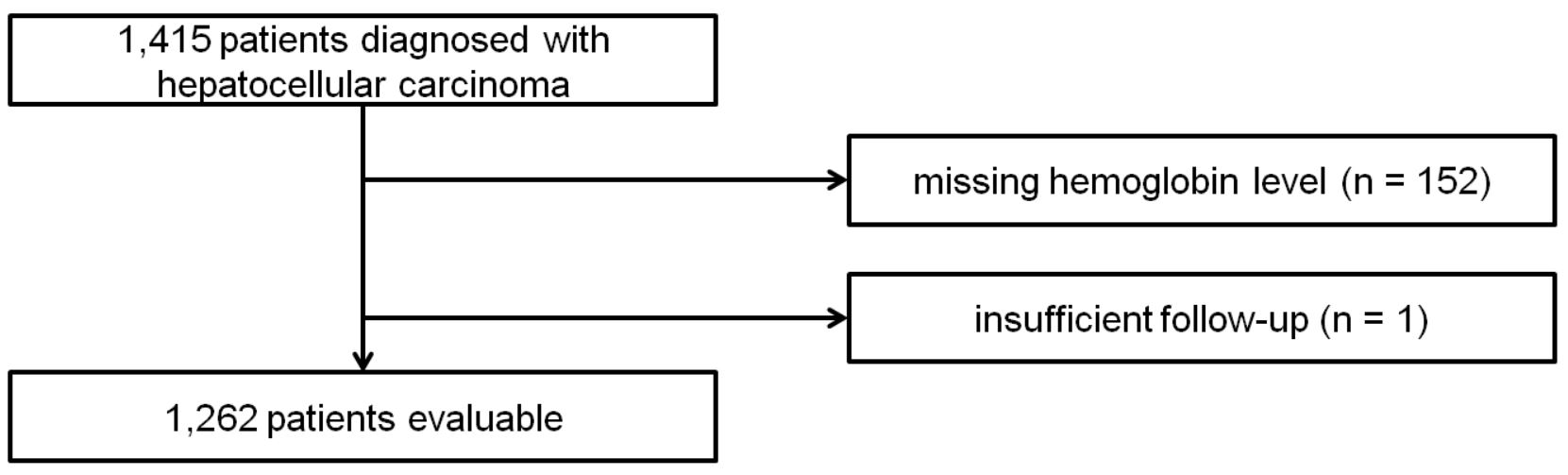


Supplementary Figure 1. Patient flow chart.


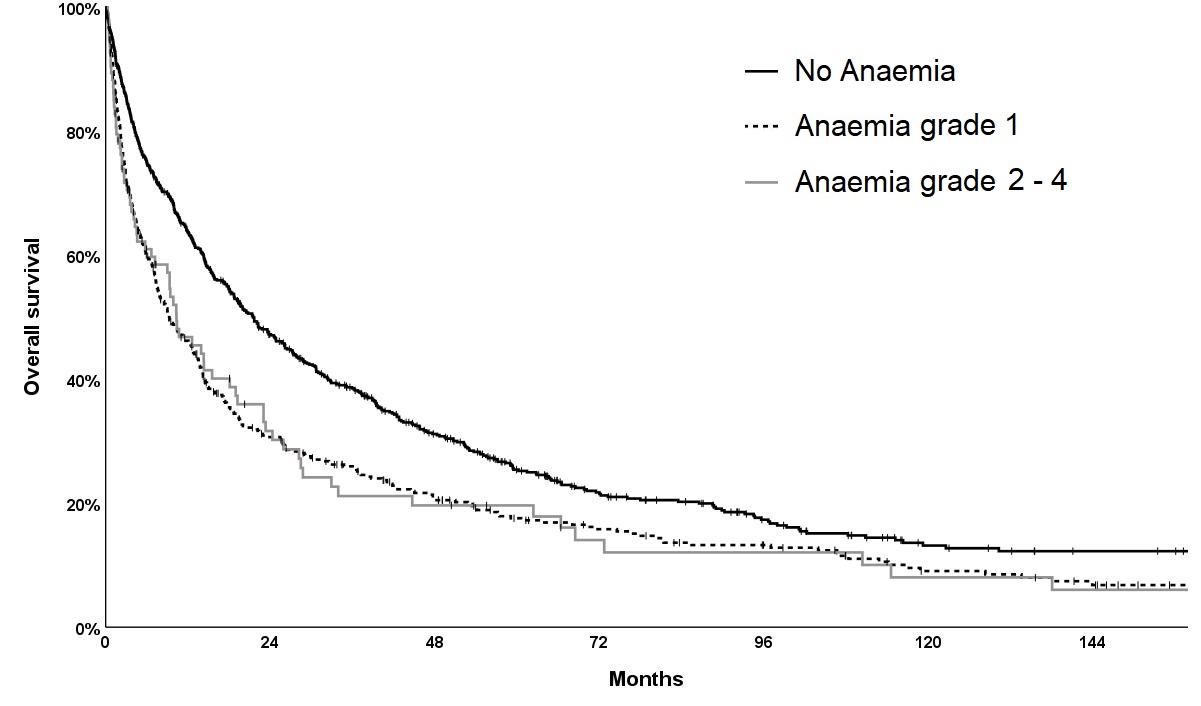


Supplementary Figure 2. Overall survival (OS) according to severity of anaemia (Common Terminology Criteria of Adverse Events version 5.0 grade).


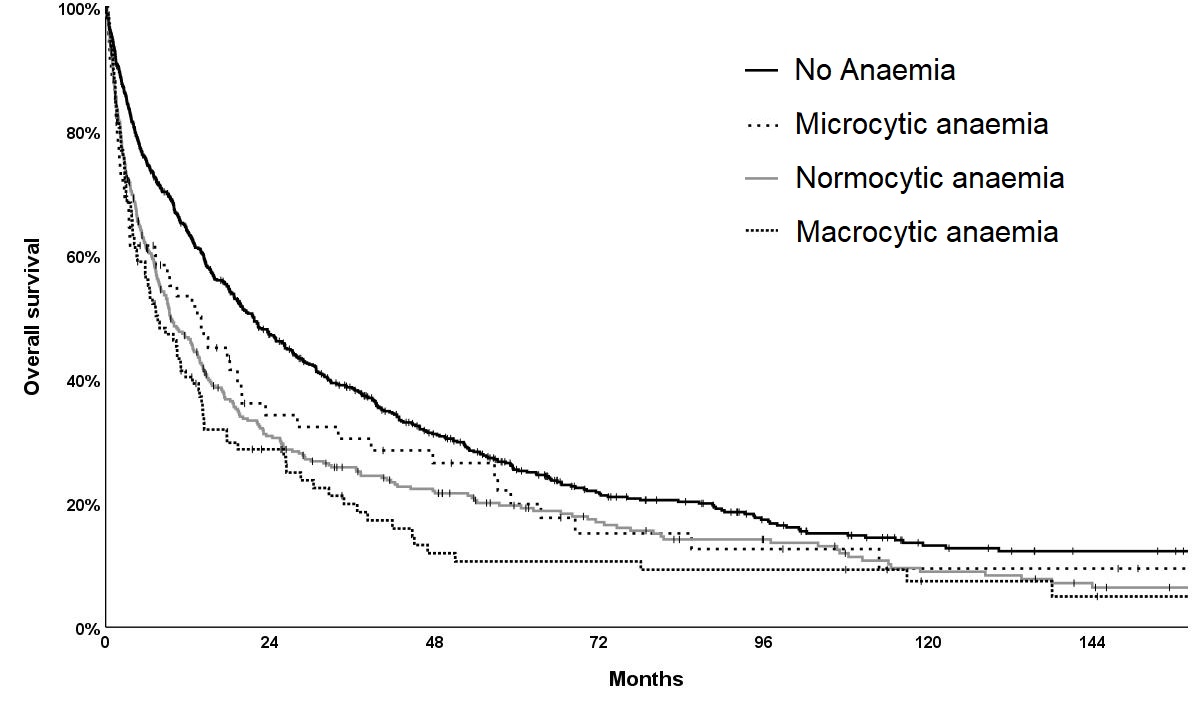


Supplementary Figure 3. Overall survival (OS) according to type of anaemia (micro-, normo-, macrocytic).


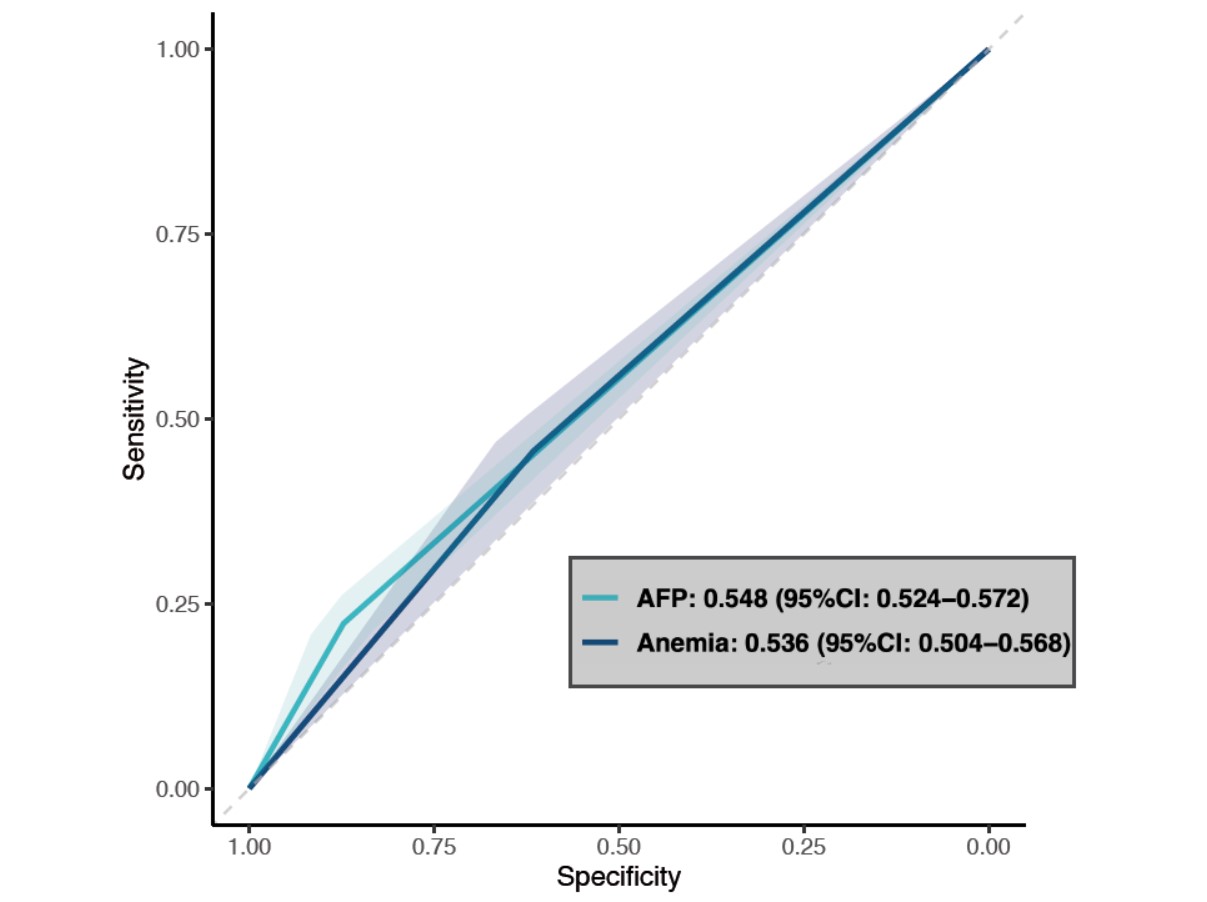


Supplementary Figure 4. Prognostic receiver operator characteristic (ROC) curves of alpha-fetoprotein (AFP; < 1000 vs. ≥ 1000 ng/mL) and anaemia (present vs. absent).
